# Complete Genome Sequence of *Salmonella enterica* Serovar Choleraesuis Vaccine Strain C500 Attenuated by Chemical Mutation

**DOI:** 10.1128/genomeA.01022-14

**Published:** 2014-10-09

**Authors:** Qiuchun Li, Yachen Hu, Lijuan Xu, Xiaolei Xie, Mingxin Tao, Xinan Jiao

**Affiliations:** Jiangsu Key Laboratory of Zoonosis/Jiangsu Co-Innovation Center for Prevention and Control of Important Animal Infectious Diseases and Zoonoses, Yangzhou University, Yangzhou, China

## Abstract

*Salmonella enterica* serovar Choleraesuis strain C500 is a live vaccine attenuated by chemical methods. Here, we report the complete genome sequence of the strain, which may be helpful for elucidating the attenuation mechanism of the vaccine strain.

## GENOME ANNOUNCEMENT

*Salmonella*, which can be transmitted from foods to humans, is the second most common bacterium causing human food-borne gastroenteritis (1). Among more than 2,500 serovars of *Salmoenlla*, some serotypes, such as Enteritidis and Typhimurium, show a broad host range and can infect various animals. Some, such as serotypes Choleraesuis and Dublin, have a narrow host range and are most highly restricted to a specific animal species (2). *Salmonella enterica* serovar Choleraesuis (*Salmonella* Choleraesuis) mainly causes paratyphoid to swine (2) but occasionally cause systemic infections in human (3). The *Salmonella* Choleraesuis reservoir in swine is a concern because of its public health implications for humans (2). Vaccination has been proven to be a feasible approach to prevent piglet paratyphoid (4). Different vaccines have been made and utilized in China, the United States, and Europe (2, 4–7).

*Salmonella* Choleraesuis strain C500 is an attenuated vaccine that has been widely used in China for over 40 years to control piglet paratyphoid (4). It has also been developed as a potential live oral vaccine vector for the delivery of DNA vaccines adapted to swine (8). However, as strain C500 was attenuated from virulent strain C78-1 by chemical methods, the genetic background of the vaccine remained unclear. In this study, whole-genome sequencing of the strain will help us to find changes of genes in the chromosome and reveal the attenuation mechanism of the vaccine.

Genomic DNA was extracted using the DNeasy blood and tissue kit (Qiagen, Germany) from strain C500 grown overnight in LB broth at 37°C with agitation, the DNA library was then constructed according to the Illumina protocol. Whole-genome sequencing was performed on an Illumina HiSeq 2000 run using a 500-bp paired-end library and a 2,000-bp paired-end library. Low-quality reads were filtered and the rest were assembled with SOAP *de novo* software (BGI, Shenzhen, China). The assembly generated 30 scaffolds consisting of 39 contigs, with a maximum scaffold length of 1,276,376 bp and a minimum length of 546 bp. All the gaps between contigs were closed manually by PCR amplification of the genomic DNA and Sanger DNA sequencing. The complete genome sequence was then submitted to the NCBI Prokaryotic Genomes Automatic Annotation Pipeline (2013).

The length of *Salmonella* Choleraesuis strain C500 is 4,751,585 bp and has a G+C content of 52.18%. There are 4,490 predicted genes in the chromosome, including 4,232 coding DNA sequences, and 151 pseudo genes, 19 rRNA genes (5S, 16S, and 23S), and 82 tRNA genes are identified in the genome. Comparison to the genome of *Salmonella* Choleraesuis strain SC-B67, the C500 strain is deficient in *rpoS* gene, a vital transcriptional regulator playing an important role in *Salmonella* infection (9, 10). This may be one of the important factors for virulence attenuation of C500 strain.

A detailed comparative analysis between genomes of C500 and SC-B67 strain will be published in a future report.

### Nucleotide sequence accession number.

The complete genome sequence for *Salmonella enterica* serovar Choleraesuis strain C500 has been deposited at GenBank under the accession number CP007639. The version described in this paper is the first version.
